# Hypoxia-Induced Autophagy Degrades Stromal Lumican into Tumor Microenvironment of Pancreatic Ductal Adenocarcinoma: A Mini-Review

**DOI:** 10.29245/2578-2967/2019/1.1165

**Published:** 2019-02-22

**Authors:** Bhaswati Sarcar, Xinqun Li, Jason B. Fleming

**Affiliations:** 1 Department of Gastrointestinal Oncology, H. Lee Moffitt Cancer Center and Research Institute, FL, USA; 2 Department of Surgical Oncology, The University of Texas MD Anderson Cancer Center, TX, USA

**Keywords:** Stellate cells, Lumican, Hypoxia, HIF-1α, p-AMPK, Autophagy

## Abstract

The extracellular matrix (ECM) in the tumor microenvironment (TME) has gained considerable interest in recent years as a crucial component in fundamental cellular processes and provides novel therapeutic targets. Lumican is a class II small leucine-rich proteoglycan with a key role in ECM organization and modulation of biological functions dependent on tumor type, abundance, and stage of disease. The presence of stromal lumican in the ECM surrounding pancreatic ductal adenocarcinoma (PDAC) inhibits cancer cell replication and is associated with improved patient outcomes after multimodal therapies. In this mini-review, were-present our novel findings describing how hypoxia (1% O2) within the TME influences stromal lumican expression and secretion. We observed that hypoxia specifically inhibited lumican expression and secretion post-transcriptionally only from pancreatic stellate cells. Hypoxia-induced increased lactate production did not influence lumican expression. Notably, autophagy was induced by hypoxia in ex vivo cultures of patient-derived primary PDAC xenograft and pancreatic stellate cells; however, the cancer cells remain unaffected. Moreover, hypoxia-inducible factor (HIF)-1α expression or inhibition of AMP-regulated protein kinase (AMPK) activation within hypoxic stellate cells restored lumican expression levels. Interestingly, AMPK inhibition attenuated hypoxia-reduced phosphorylation of the mTOR/p70S6K/4EBP signaling pathway. The aim of this mini-review is to summarize our recent publication that hypoxia reduces stromal lumican in PDAC through autophagy-mediated degradation and reduction in protein synthesis within pancreatic cancer stellate cells. This may provide another plausible mechanism by which hypoxia-induced stromal autophagy leads to cancer growth.

## Pancreatic ductal adenocarcinoma and Lumican

Pancreatic ductal adenocarcinoma (PDAC) is a very aggressive malignancy with a high incidence of distant metastasis^1^ and is projected to be the second leading cause of cancer death by 2030^2^. The pathways that promote invasion and metastasis of PDAC are elusive. However, elucidating the mechanisms behind the metastatic efficiency of this deadly disease is important to develop a novel therapeutic approach. PDAC exhibits extensive desmoplastic stroma, which derives from activated pancreatic stellate cells. This activation results in proliferation, leading to the production of collagen, laminin, fibronectin^3^, and lumican^4^ within the extracellular matrix (ECM). Lumican, a member of a class II family of small leucine-rich proteoglycans, is highly upregulated in different cancer types, especially pancreas, breast, and cervical cancer^5–8^, and affects the proliferation, migration, and adhesion of cancer cells through a variety of mechanisms^4,9–13^. Studies have demonstrated differential distribution of lumican between cancer cells and the reactive tumor stroma^4,13–16^. In pancreatic cancer, lumican is specifically localized in alpha cells of islets, acinar cells, collagen fibrils, fibroblasts close to pancreatic cancer cells, and cancer cells^5^. Our previous report and others’ confirm that pancreatic stellate cells are the major source of lumican production and secretion^4,6^. Recently, it has been shown that heat shock protein 47 (HSP47) interacts with and promotes the secretion of lumican in the ECM^17^. Lumican was shown to influence cell function through various mechanisms in a tissue-specific manner in different cancers. In the tumor microenvironment (TME) of primary PDAC, the presence of lumican within the ECM has been positively and negatively correlated with tumor progression. It has previously been reported that the localization of lumican in the stromal tissue correlates with advanced-stage pancreatic cancer, retroperitoneal and duodenal invasion, and tends to correlate with shorter survival^18^. Degradation of lumican by matrix metalloproteinases MTI-MMP resulted in the abrogation of p21-mediated suppression of tum origenicity by lumican^19^. On the other hand, a recent study has demonstrated that glycosylated lumican possesses anti-tumor activity by directly interacting with the catalytic domain of M M P-14 and inhibiting its activity^20^. It was reported that lumican manifested anticancer activity in invasive breast cancer cells by modifying cell morphology, evoking EMT/MET reprogramming, and suppressing matrix metalloproteinases and epidermal growth factor receptors (EGFRs)^21^. Conversely, in the gastric cancer-associated fibroblasts, lumican activated the (β1 integrin-m ediated FAK signaling, thereby enhancing tumor grow th^22^. Moreover, secreted 70-kDa lumican by PDAC cells stimulated growth and inhibited invasion of human pancreatic cancer. It also activated ERK signaling, induced a 3 integrin expression, and decreased active matrix metalloproteinase-9^23^. On the contrary, lumican overexpression within the pancreatic cancer TME in vivo produced uniformly smaller tumors, correlated with reduced vascular density, enhanced Fas-mediated endothelial apoptosis, and reduced angiogenesis in TME^24,25^. A key finding in our previous study was that the activated pancreatic stellate cells within the TME are the principle source of stromal lumican, and the presence of lumican within the stroma of a primary PDAC tumor is associated with decreased metastasis and prolonged survival in early-stage pancreatic cancer^12,13^. Mechanistically, we demonstrated that the activated pancreatic stellate cells within PDAC secrete lumican under the negative control of TGF-β and enhance stellate cell adhesion and mobility in a collagen-rich environment^4^. We also demonstrated that extracellular lumican physically binds with EGFRs to trigger EGFR internalization, downregulation of EGFR, and its downstream signal. This resulted in apoptosis, glycolytic metabolism inhibition, and entry into a quiescent state^12,26^. Mounting evidence reported in multiple studies on PDAC by our group^4,12,13,26^ and several others using different cancer m odels^15,27–30^ suggests that lumican is a key regulator of ECM and ECM-cell interactions within the TME. Overall, lumican is shown to have an anti-tumor role in a context-dependent manner, and the conflicting results regarding the precise role of lumican in tumor progression and regression depend on the specific correlation between lumican tissue-specific abundance, distribution, and stage of disease in PDAC.

## Hypoxia Attenuates the Secretion of Lumican by Pancreatic Stellate Cells

**Figure 1:**
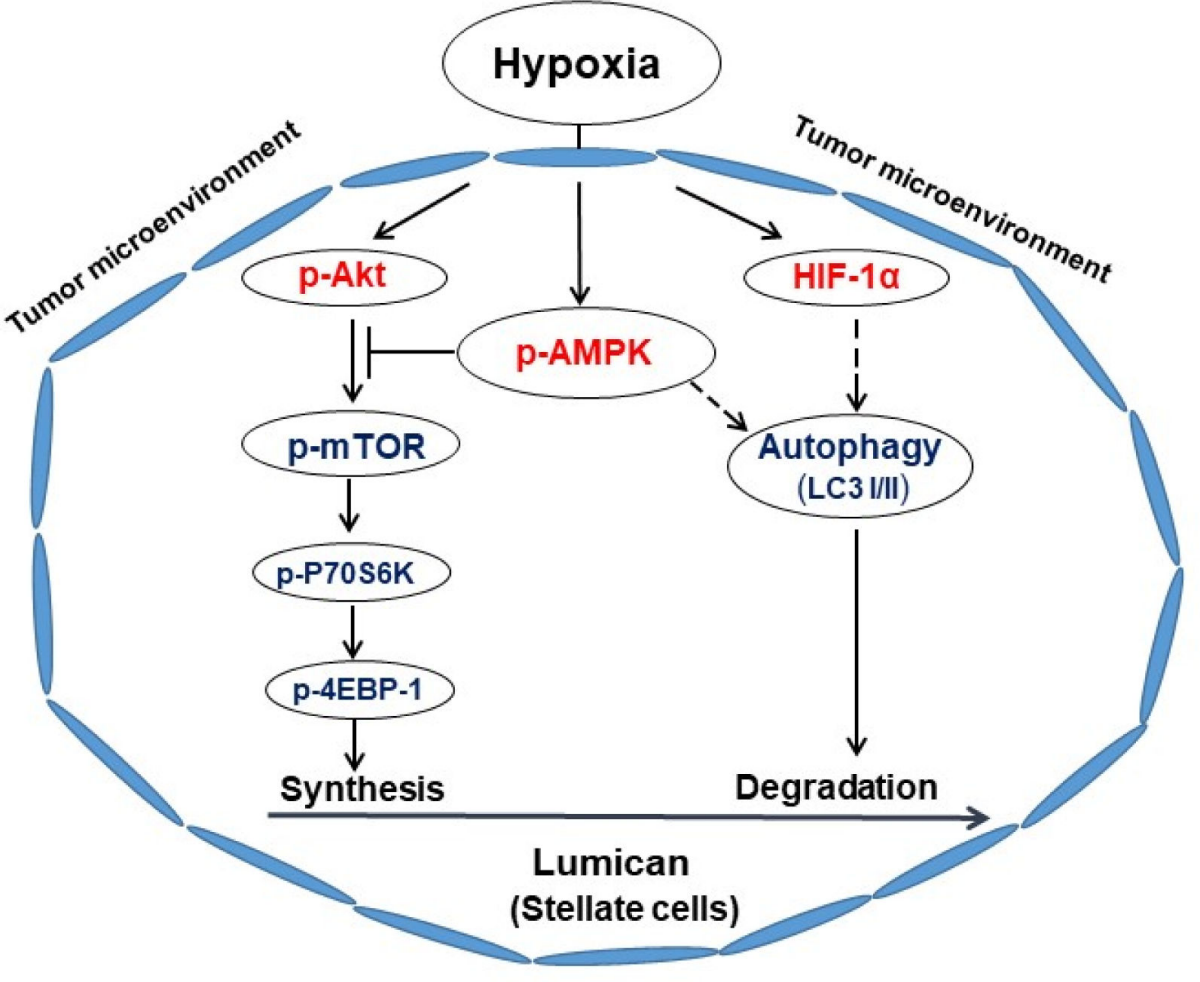


The PDAC TME is characterized by dense desmoplastic stroma and resultant hypoxia, which also drives angiogenesis, immune suppression, and numerous signaling events that promote cancer progression, metastasis, and poor patient survival.^31–34^ The influence of hypoxia on stromal composition and secreted extracellular protein such as lumican is not clearly understood. Therefore, in our recent publication^35^, our major focus was to investigate the extent to which hypoxia influences stromal expression and secretion of lumican in PDAC. We observed that hypoxia significantly reduced lumican expression and secretion from pancreatic stellate cells but not cancer cells. Our results revealed that hypoxia stimulates HIF-1α and AMP-regulated protein kinase (AMPK) activation, which leads to autophagy and subsequent reduction in cellular and secreted lumican (Figure 1). We found that AMPK activation blocked the hypoxia-activated mTOR signaling pathway, which in turn inhibited lumican protein synthesis (Figure 1). Therefore, the hypoxia-activated cell signaling event reduces the level of lumican production by stellate cells in the ECM of PDAC.

Desmoplastic fibrosis in PDAC tumors inhibits the delivery of nutrients and oxygen, which produces a TME that is characterized by hypoxia and metabolic stress. We expanded our study^35^ by applying a robust ex vivo organotypic live-tissue culture system to mimic tumor-stroma coevolution and provide a hypoxic environment that mimics the TME. The culture system consisted of precision-cut and uniform small tissue slices (200^m) derived from patient-derived primary PDAC xenograft (PDX) tumors. Importantly, the tissue slices remained viable for at least 5 days. In our previous study, we have shown that microenvironment components, tumor architecture, and cell signaling pathways^36^ are well preserved in those tumor tissue slices. The slices were cultured in hypoxia (1% O2) for up to 48 hrs. and this unique ex vivo system allowed for the measurement of autophagic changes within the stroma and the reduction of stromal lumican that is exposed to hypoxic conditions. We went on to confirm the finding that hypoxia induces autophagy and attenuates lumican secretion by conducting in vitro studies using activated human stellate cells (HPSCs and HPaSTeC) cultured from human PDAC tumors.

## Hypoxia-Induced Autophagy-Mediated Degradation of Lumican in Tumor Stroma

Cellular protein degradations are governed by 2 major pathways in the eukaryotic system: the ubiquitinproteosome system (UPS) and autophagy. The latter is primarily responsible for the degradation of aggregated proteins and cellular organelles^37^. The hypoxia and metabolic stress within the TME aggravated cellular autophagy. Previous autophagy-related studies focused only on cancer cells^38,39^ but recent advancements in research on autophagy have led to a focus on the tumor stroma, preferably referred to as the “autophagic tumor stroma,” which is an adaptation process where the autophagy of stromal cells provides fuel for cancer cells within the TME^39–41^. Next, to determine which pathway is involved in the lumican degradation process, stellate cells were treated with a UPS inhibitor and autophagy-lysosomal inhibitors in the normoxic and hypoxic environments. Our data indicated that hypoxia reduced stromal lumican via autophagy, not the UPS pathway. We went on to confirm this finding by knocking down proautophagic signaling gene Beclin 1 (Atg6)^42^ by using RNA interference. Silencing of Beclin 1 set back the hypoxia-induced decrease in lumican that is noticed in stellate cells. We also confirmed the cellular colocalization of the lumican and lysosomal marker LAMP in hypoxic stellate cells. This indicated that lumican is predominantly transported to the lysosome when stellate cells are hypoxic. It is well accepted that hypoxia initiates tumor cell autophagy in different cancers^43–45^. In our recent study^35^, we sought to determine whether hypoxia promotes autophagy within pancreatic stellate cells, and our investigation resulted in increasing expressions of the canonical autophagic marker LC3 in both stellate cells and PDX slices that were cultured selectively in hypoxic conditions. Our observations suggested that hypoxia induces autophagy, which results in the degradation of lumican within PDAC stellate cells.

## Activated AMPK Negatively Regulates Hypoxia-Induced Stromal Lumican Synthesis

HIF-1α and the proautophagic module AMPK have been known to influence hypoxia-induced autophagy^46–48^. Inquisitively, we have investigated their role in mediating hypoxia-induced autophagy in pancreatic stromal cells. We evaluated the colocalizations of lumican with HIF-1α and phosphorylated AMPK at the interface between cancer cells and stromal cells in patient tumor tissues. Hypoxia stimulated AMPK activation and the increased expressions of HIF-1α and LC3 in vitro and ex vivo. Treatments consisting of pharmacological AMPK inhibitors and siRNA knockdown of HIF-1 α both inhibited hypoxia-induced reduction of lumican expressions. Intriguingly, to define the role of HIF-1α, we transfected the stellate cells with w ild type and mutant HIF-1 α construct. We noticed that the native HIF-1α, not the mutant, is required for lumican degradation in stromal cells. Therefore, hypoxia-related HIF-1α and AMPK activation mediated the induction of autophagy in the stroma, resulting in lumican degradation. Mechanistically, we found that hypoxia does not influence lumican protein synthesis transcriptionally. We next targeted PI3K/Akt/mTOR pathways and their downstream signaling in stromal cells and in PDX slices in hypoxia to examine the involvement of translational machinery related to lumican protein synthesis. Interestingly, Akt phosphorylation was significantly increased, whereas its remaining antiautophagic node signals, mTOR, P70S6K, and 4-EBP-1 (Figure 1), were decreased during exposure to hypoxia. This finding is concomitant with the hypoxia-enhanced AM PK activation. Previous literature has suggested that Akt-mediated mTOR signaling can be blocked by activated pro-autophagic signaling molecule AMPK, which phosphorylates TSC2^49,50^. Interestingly, treatment of stellate cells with the AMPK inhibitor reversed the hypoxia-related reduction in the mTOR pathway and its downstream signaling (Figure 1). Moreover, it also enhanced the lumican expression transcriptionally, which indicated that hypoxia-induced mTOR activation was blocked by activated AMPK. Therefore, molecular mechanistic studies by our group demonstrated that activated AM PK in hypoxic stellate cells negatively impacts lumican protein synthesis.

## Downregulation of Stromal Lumican is not Influenced by Increased Lactate Production

Nearly a century ago, Warburg and his colleague made the seminal observation that the common feature of cancer cell metabolism is increased glucose uptake and the fermentation of glucose into lactate to promote growth, survival, proliferation, and long-term maintenance^51^. The lactate can be transferred to other cells through specialized transporters, which provides another fuel source for oxidative mitochondrial metabolism^52,53^. Recently, stromal cells have been recognized as a lactate source for use by cancer cells^54^. Therefore, in this context, we examined whether stromal lactate, triggered by hypoxia, influenced the observed reduction of lumican in TME. We found that the presence or absence of lactate did not influence lumican expression, although hypoxia dramatically enhanced the lactate dehydrogenase A (LDHA) expression and secretion of lactate in stromal cells. Our future study will assess the expression of a lactate transporter, monocarboxylate transporter 1 (MCT1), and the activation of AMPK and HIF-1α in PDAC stromal cells to gain a better insight into lactate shuttling from PDAC to its stromal cells.

## Conclusions

This review summarizes our recent publication^35^ addressing the factors within the TME that influence lumican production in stromal cells. We consistently found that 1) hypoxia-induced autophagy in PDAC stellate cells; 2) subsequent lumican degradation occurred exclusively in stromal cells, leaving the cancer cells, indicating that stellate cells are more sensitive to hypoxia with respect to lumican levels; and 3) stromal autophagy decreased stromal lumican secretion, which was linked to tumor growth^12,13^. In conclusion, these recent findings by our group provide important new insights to build and deepen our understanding of the molecular interactions between stromal lumican and the TME and provide a rationale to design credible antitumor approaches against pancreatic cancer.

## References

[1] Iozzo RV. The family of the small leucine-rich proteoglycans: key regulators of matrix assem bly and cellular growth. Crit Rev Biochem Mol Biol. 1997; 32(2): 141–174. 9145286 10.3109/10409239709108551

[2] Rahib L, Smith BD, Aizenberg R, Projecting cancer incidence and deaths to 2030: the unexpected burden of thyroid, liver, and pancreas cancers in the United States. Cancer Res. 2014; 74(11): 2913–2921. 24840647 10.1158/0008-5472.CAN-14-0155

[3] Apte MV, Wilson JS, Lugea A, A starring role for stellate cells in the pancreatic cancer microenvironment. Gastroenterology. 2013; 144(6): 1210–1219. 23622130 10.1053/j.gastro.2012.11.037 PMC3729446

[4] Kang Y, Roife D, Lee Y, Transforming Growth Factor-beta Lim its Secretion of Lumican by Activated Stellate Cells within Primary Pancreatic Adenocarcinoma Tumors. Clin Cancer Res. 2016; 22(19): 4934–4946. 27126993 10.1158/1078-0432.CCR-15-2780 PMC5127652

[5] Ping Lu Y, Ishiwata T, Asano G. Lumican expression in alpha cells of islets in pancreas and pancreatic cancer cells. J Pathol. 2002; 196(3): 324–330. 11857496 10.1002/path.1037

[6] Koninger J, Giese T, di Mola FF, Pancreatic tum or cells influence the com position of the extracellular matrix. Biochem Biophys Res Commun. 2004; 322(3): 943–949. 15336555 10.1016/j.bbrc.2004.08.008

[7] Leygue E, Snell L, Dotzlaw H, Expression of lumican in human breast carcinoma. Cancer Res. 1998; 58(7): 1348–1352. 9537227

[8] Naito Z, Ishiwata T, Kurban G, Expression and accumulation of lumican protein in uterine cervical cancer cells at the periphery of cancer nests. Int J Oncol. 2002; 20(5): 943–948. 11956587

[9] D ‘Onofrio MF, Brezillon S, Baranek T, Identification of be tal integrin as mediator of melanom a cell adhesion to lumican. Biochem Biophys Res Commun. 2008; 365(2): 266–272. 17981144 10.1016/j.bbrc.2007.10.155

[10] Seomun Y, Joo CK. Lumican induces human corneal epithelial cell migration and integrin expression via ERK 1/2 signaling. Biochem Biophys Res Commun. 2008; 372(1): 221–225. 18477477 10.1016/j.bbrc.2008.05.014

[11] Kang Y, Zhang R, Suzuki R, Two-dimensional culture of human pancreatic adenocarcinoma cells results in an irreversible transition from epithelial to mesenchymal phenotype. Lab Invest. 2015; 95(2): 207–222. 25485535 10.1038/labinvest.2014.143 PMC4670045

[12] Li X, Kang Y, Roife D, Prolonged exposure to extracellular lumican restrains pancreatic adenocarcinom a growth. Oncogene. 2017; 36(38): 5432–5438. 28534517 10.1038/onc.2017.125 PMC5799806

[13] Li X, Tru ty MA, Kang Y, Extracellular lumican inhibits pancreatic cancer cell growth and is associated with prolon ged survival after surgery. Clin Cancer Res. 2014; 20(24): 6529–6540. 25336691 10.1158/1078-0432.CCR-14-0970 PMC4268437

[14] Nikitovic D, Katonis P, Tsatsakis A, Lumican, a small leucine-rich proteoglycan. IUBMB Life. 2008; 60(12): 818–823. 18949819 10.1002/iub.131

[15] Brezillon S, Pietraszek K, Maquart FX, Lumican effects in the control of tum our progression and their links with metalloproteinases and integrins. FEBS J. 2013; 280(10): 2369–2381. 23438179 10.1111/febs.12210

[16] Wegrowski Y, Maquart FX. Involvement of stromal proteoglycans in tum our progression. Crit Rev Oncol Hematol. 2004; 49(3): 259–268. 15036265 10.1016/j.critrevonc.2003.10.005

[17] Ishikawa Y, Rubin K, Bachinger HP, The endoplasmic reticulum-resident collagen chaperone Hsp47 interacts with and promotes the secretion of decorin, fibromodulin and lumican. J Biol Chem. 2018. 10.1074/jbc.RA117.000758 PMC6120207 30002123

[18] Ishiwata T, Cho K, Kawahara K, Role of lumican in cancer cells and adjacent strom al tissues in human pancreatic cancer. Oncol Rep. 2007; 18(3): 537–543. 17671699

[19] Li Y, Aoki T, Mori Y, Cleavage of lumican by membrane-type matrix m etalloproteinase-1 abrogates this proteoglycan-mediated suppression of tum or cell colony form ation in soft agar. Cancer Res. 2004; 64(19): 7058–7064. 15466200 10.1158/0008-5472.CAN-04-1038

[20] Pietraszek-Gremplewicz K, Karamanou K, Niang A, Small leucine-rich proteoglycans and matrix metalloproteinase-14: Key partners? Matrix Biol. 2019; 75–76: 271–285. 10.1016/j.matbio.2017.12.006 29253518

[21] Karamanou K, Franchi M, Piperigkou Z, Lumican effectively regulates the estrogen receptors-associated functional properties of breast cancer cells, expression of matrix effectors and epithelial-to-mesenchymal transition. Sci Rep. 2017; 7: 45138. 28332606 10.1038/srep45138 PMC5362815

[22] Wang X, Zhou Q, Yu Z, Cancer-associated fibroblast-derived Lumican promotes gastric cancer progression via the integrin beta1-FAK signaling pathway. Int J Cancer. 2017; 141(5): 998–1010. 28542982 10.1002/ijc.30801

[23] Yamamoto T, Matsuda Y, Kawahara K, Secreted 70kDa lumican stimulates growth and inhibits invasion of human pancreatic cancer. Cancer Lett. 2012; 320(1): 31–39. 22266188 10.1016/j.canlet.2012.01.023

[24] William s KE, Fulford LA, A lbig AR. Lumican reduces tum or growth via induction of fas-mediated endothelial cell apoptosis. Cancer Microenviron. 2010; 4(1): 115–126. 21505566 10.1007/s12307-010-0056-1 PMC3047633

[25] Sharma B, Ramus MD, K irk wood CT, Lumican exhibits anti-angiogenic activity in a context specific manner. Cancer Microenviron. 2013; 6(3): 263–271. 23775523 10.1007/s12307-013-0134-2 PMC3855379

[26] Li X, Roife D, Kang Y, Extracellular lumican augments cytotoxicity of chemotherapy in pancreatic ductal adenocarcinom a cells via autophagy inhibition. Oncogene. 2016; 35(37): 4881–4890. 26876211 10.1038/onc.2016.20

[27] Brezillon S, Radwanska A, Zeltz C, Lumican core protein inhibits melanoma cell migration via alterations of focal adhesion complexes. Cancer Lett. 2009; 283(1): 92–100. 19394140 10.1016/j.canlet.2009.03.032

[28] Brezillon S, Venteo L, Ram ont L, Expression of lumican, a small leucine-rich proteoglycan with antitum our activity, in human malignant melanom a. Clin Exp Dermatol. 2007; 32(4): 405–416. 17490399 10.1111/j.1365-2230.2007.02437.x

[29] Brezillon S, Zeltz C, Schneider L, Lumican inhibits B16F1 melanoma cell lung metastasis. J Physiol Pharm acol. 2009; 60 Suppl 4: 15–22. 20083847

[30] Gore J, Korc M. Pancreatic cancer stroma: friend or foe? Cancer Cell. 2014; 25(6): 711–712. 24937454 10.1016/j.ccr.2014.05.026 PMC4821630

[31] Buchler P, Reber HA, Lavey RS, Tum or hypoxia correlates with metastatic tum or growth of pancreatic cancer in an orthotopic murine model. J Surg Res. 2004; 120(2): 295–303. 15234226 10.1016/j.jss.2004.02.014

[32] Bao B, Ali S, Ahmad A, Hypoxia-induced aggressiveness of pancreatic cancer cells is due to increased expression of VEGF, IL-6 and miR-21, which can be attenuated by CDF treatment. PLoS One. 2012; 7(12): e50165. 23272057 10.1371/journal.pone.0050165 PMC3521759

[33] Farazi TA, Spitzer JI, Morozov P, miRNAs in human cancer. J Pathol. 2011; 223(2): 102–115. 21125669 10.1002/path.2806 PMC3069496

[34] Greither T, Grochola LF, Udelnow A, Elevated expression of microRNAs 155, 203, 210 and 222 in pancreatic tumors is associated with poorer survival. Int J Cancer. 2010; 126(1): 73–80. 19551852 10.1002/ijc.24687

[35] Li X, Lee Y, Ya Kang, Hypoxia-induced autophagy of stellate cells inhibits expression and secretion of lumican into microenvironment of pancreatic ductal adenocarcinoma. Cell Death & D ifferentiation. 2018. 10.1038/s41418-018-0207-3 PMC6329841 30283082

[36] Roife D, Dai B, Kang Y, Ex Vivo Testing of Patient-Derived Xenografts Mirrors the Clinical Outcome of Patients with Pancreatic Ductal Adenocarcinoma. Clin Cancer Res. 2016; 22(24): 6021–6030. 27259561 10.1158/1078-0432.CCR-15-2936 PMC5136340

[37] Lilienbaum A Relationship between the proteasomal system and autophagy. Int J Biochem MolBiol. 2013; 4(1): 1–26. PMC3627065 23638318

[38] Apel A, Zentgraf H, Buchler MW, Autophagy-A double-edged sword in oncology. Int J Cancer. 2009; 125(5): 991–995. 19452527 10.1002/ijc.24500

[39] Yang Z, Klionsky DJ. Eaten alive: a history of macroautophagy. Nat Cell Biol. 2010; 12(9): 814–822. 20811353 10.1038/ncb0910-814 PMC3616322

[40] Levine B, Kroemer G. Autophagy in the pathogenesis of disease. Cell. 2008; 132(1): 27–42. 18191218 10.1016/j.cell.2007.12.018 PMC2696814

[41] Shintani T, Klionsky DJ. Autophagy in health and disease: a double-edged sword. Science. 2004; 306(5698): 990–995. 15528435 10.1126/science.1099993 PMC1705980

[42] Mizushim a N, Levine B. Autophagy in mammalian development and differentiation. Nat Cell Biol. 2010; 12(9): 823–830. 20811354 10.1038/ncb0910-823 PMC3127249

[43] Cao Y, Luo Y, Zou J, Autophagy and its role in gastric cancer. Clin Chim Acta. 2018; 489: 10–20. 30472237 10.1016/j.cca.2018.11.028

[44] Zhang J, Chu D, Kawamura T, GRIM-19 repressed hypoxia-induced invasion and EMT of colorectal cancer by repressing autophagy through inactivation of STAT3/HIF-1alpha signaling axis. J Cell Physiol. 2018. 10.1002/jcp.27914 PMC13483207 30537081

[45] Huang S, Qi P, Zhang T, The HIF1alpha/m iR2243p/ATG5 axis affects cell mobility and chemosensitivity by regulating hypoxiainduced protective autophagy in glioblastom a and astrocytom a. Oncol Rep. 2018. 10.3892/or.2018.6929 30569180

[46] Zhang H, Bosch-Marce M, Shimoda LA, Mitochondrial autophagy is an HIF-1-dependent adaptive metabolic response to hypoxia. J Biol Chem. 2008; 283(16): 10892–10903. 18281291 10.1074/jbc.M800102200 PMC2447655

[47] Gozuacik D, Kimchi A. Autophagy as a cell death and tum or suppressor mechanism. Oncogene. 2004; 23(16): 2891–2906. 15077152 10.1038/sj.onc.1207521

[48] Jaattela M Multiple cell death pathways as regulators of tum our initiation and progression. Oncogene. 2004; 23(16): 2746–2756. 15077138 10.1038/sj.onc.1207513

[49] Huang J, Manning BD. A com plex interplay between Akt, TSC2 and the two mTOR complexes. Biochem Soc Trans. 2009; 37(P t 1): 217–222. 19143635 10.1042/BST0370217 PMC2778026

[50] Chen W, Pan Y, Wang S, Cryptotanshinone activates AMPK-TSC2 axis leading to inhibition of mTORC1 signaling in cancer cells. BMC Cancer. 2017; 17(1): 34. 28061838 10.1186/s12885-016-3038-y PMC5219700

[51] Warburg O, Wind F, Negelein E. The Metabolism of Tumors in the Body. J Gen Physiol. 1927; 8(6): 519–530. 19872213 10.1085/jgp.8.6.519 PMC2140820

[52] Gladden LB. Alactatic perspective on metabolism. Med Sci Sports Exerc. 2008; 40(3): 477–485. 18379210 10.1249/MSS.0b013e31815fa580

[53] Brooks GA. Lactate: link between glycolytic and oxidative metabolism. Sports Med. 2007; 37(4–5): 341–343. 17465603 10.2165/00007256-200737040-00017

[54] Martinez-Outschoorn UE, Balliet RM, Rivadeneira DB, Oxidative stress in cancer associated fibroblasts drives tum or-stroma co-evolution: A new paradigm for understanding tumor metabolism, the field effect and genomic instability in cancer cells. Cell Cycle. 2010; 9(16): 3256–3276. 20814239 10.4161/cc.9.16.12553 PMC3041164

